# Murine Lung Responses to Ambient Particulate Matter: Genomic Analysis and Influence on Airway Hyperresponsiveness

**DOI:** 10.1289/ehp.11229

**Published:** 2008-06-20

**Authors:** Ting Wang, Liliana Moreno-Vinasco, Yong Huang, Gabriel D. Lang, Jered D. Linares, Sascha N. Goonewardena, Alayna Grabavoy, Jonathan M. Samet, Alison S. Geyh, Patrick N. Breysse, Yves A. Lussier, Viswanathan Natarajan, Joe G.N. Garcia

**Affiliations:** 1 Section of Pulmonary and Critical Care Medicine, University of Chicago, Chicago, Illinois, USA; 2 Section of Genetic Medicine, Department of Medicine, University of Chicago, Chicago, Illinois, USA; 3 Department of Epidemiology, Johns Hopkins University, Baltimore, Maryland, USA; 4 Department of Environmental Health Sciences, Johns Hopkins Bloomberg School of Public Health, Johns Hopkins University, Baltimore, Maryland, USA

**Keywords:** airway hyperresponsiveness, asthma, intelectin, particulate matter, toxicogenomics

## Abstract

**Background:**

Asthma is a complex disease characterized by airway hyperresponsiveness (AHR) and chronic airway inflammation. Epidemiologic studies have demonstrated that exposures to environmental factors such as ambient particulate matter (PM), a major air pollutant, contribute to increased asthma prevalence and exacerbations.

**Objective:**

We investigated pathophysiologic responses to Baltimore, Maryland, ambient PM (median diameter, 1.78 μm) in a murine model of asthma and attempted to identify PM-specific genomic/molecular signatures.

**Methods:**

We exposed ovalbumin (OVA)-sensitized A/J mice intratracheally to PM (20 mg/kg), and assayed both AHR and bronchoalveolar lavage (BAL) on days 1, 4, and 7 after PM exposure. Lung gene expression profiling was analyzed in OVA- and PM-challenged mice.

**Results:**

Consistent with this murine model of asthma, we observed significant increases in airway responsiveness in OVA-treated mice, with PM exposure inducing significant changes in AHR in both naive mice and OVA-induced asthmatic mice. PM evoked eosinophil and neutrophil infiltration into airways, elevated BAL protein content, and stimulated secretion of type 1 T helper (T_H_1) cytokines [interferon-γ, interleukin-6 (IL-6), tumor necrosis factor-α] and T_H_2 cytokines (IL-4, IL-5, eotaxin) into murine airways. Furthermore, PM consistently induced expression of genes involved in innate immune responses, chemotaxis, and complement system pathways.

**Conclusion:**

This study is consistent with emerging epidemiologic evidence and indicates that PM exposure evokes proinflammatory and allergic molecular signatures that may directly contribute to the asthma susceptibility in naive subjects and increased severity in affected asthmatics.

Extensive epidemiologic research confirms the association between increasing cardio-pulmonary morbidity and mortality and short-term exposure to ambient particulate air pollution (Dominici et al. 2003; Samet and Krewski 2007). Although the relative risk estimates are small, public health concerns exist because of the large population under exposure and the existence of high-risk groups, such as the elderly, diabetics, and those with cardiopulmonary diseases (Johnson and Graham 2005). Increase in particulate air pollution levels are associated with increased hospital admissions and emergency department visits for respiratory diseases, such as asthma and chronic obstructive pulmonary disease (COPD) (Brunekreef and Forsberg 2005). Unfortunately, despite significant epidemiologic evidence, experimental studies of animals and human exposure to inhaled concentrated ambient particulate matter (PM) have yet to provide substantial insights into specific mechanisms of the pathophysiologic consequences of exposure to ambient PM (Handzel 2000; Peden 2001).

Preclinical animal models have been used to examine the relationship between exposure to PM and airway pathogenesis and elevated airway hyperresponsiveness (AHR) (Archer et al. 2004; de Haar et al. 2006; Walters et al. 2001) both in naive animals and in animals sensitized by antigen challenge (Fernvik et al. 2002; Steerenberg et al. 2003). However, it remains unclear as to which mechanisms are involved in producing local airway damage and adjuvant activity to antigen challenge. To address potential mechanisms of PM-mediated AHR in both control and at-risk populations, we employed a murine model of AHR and asthma induced by sensitization to ovalbumin (OVA). We exposed naive and OVA-sensitized mice to Baltimore, Maryland, ambient PM, a challenge we previously demonstrated to produce acute airway toxicity (Walters et al. 2001). In addition, we applied genomic strategies to characterize acute PM pulmonary effects in OVA-challenged mice in order to define molecular signatures produced by PM, an approach successfully employed in other inflammatory lung disorders (Girgis et al. 2005; Grigoryev et al. 2004; Ma et al. 2005; Nonas et al. 2007; Simon et al. 2006). We now report that acute ambient PM exposure induces significant changes in AHR, accompanied by eosinophil/neutrophil infiltration and type 1 T helper (T_H_1)/T_H_2 cytokine secretion in both naive mice and OVA-induced asthmatic mice. These studies indicate that the pathophysiologic effects of PM, validated in an OVA-challenged murine model of asthma, may directly contribute to the asthma susceptibility in naive subjects and increased severity in affected asthmatics.

## Materials and Methods

### Baltimore PM generation

The collection and characterization of ambient Baltimore PM used in these studies have been previously described (Walters et al. 2001, 2002). Briefly, PM was collected from a sixth floor window in urban Baltimore using a high-volume cyclone collector (theoretical cut point of 0.85 μm aerodynamic diameter) intermittently operated over a period of months with a flow rate of 0.6 m^3^/min. Collected PM was pooled and refrigerated until use. The count median diameter of PM was 1.78 μm with a geometric standard deviation of 2.21.

### Murine model of asthma

Male A/J mice (8–12 weeks of age; Jackson Laboratories, Bar Harbor, ME) were housed in an environmentally controlled animal facility at the University of Chicago for the duration of the experiments. All animal procedures conformed to the principles for laboratory animal research outlined by the Animal Welfare Act (1966) and the National Institutes of Health guidelines for the experimental use of animals (Institute of Laboratory Animal Resources 1996), as well as the common guideline of the University of Chicago Animal Care and Use Committee. All procedures were designed to treat the animals humanely and with regard for the alleviation of suffering.

Experimental asthma was induced in 8- to 12-week-old A/J mice by OVA sensitization [0.4 mg/kg, intraperitoneally (ip); day –17) and an intratracheal OVA challenge (30 mg/kg, day –3) as previously described (Ewart et al. 2000). PM was delivered 3 days post-OVA challenge (day 0) via intratracheal instillation, as previously described (Walters et al. 2001, 2002) into anesthetized mice (45 mg/kg ketamine and 8 mg/kg xylazine) suspended on a 60° incline board. With the tongue gently extended, a 50-μL aliquot of PM suspended in phosphate-buffered saline (PBS; 20 mg/kg), was placed in the back of the oral cavity and aspirated by the animal. We designed four experimental groups: PBS/PBS (all OVA and PM treatments replaced with PBS), PBS/PM (OVA treatments replaced with PBS), OVA/PBS (PM treatment replaced with PBS), and OVA/PM (received both OVA and PM treatments). AHR was determined after PM exposure of 1, 4, or 7 days and animals were sacrificed for bronchoalveolar lavage (BAL) extraction and tissue harvesting.

### Airway responsiveness measurements

We assessed airway responsiveness to intravenously administered acetylcholine (ACh) as previously described (Moreno et al. 2003). Mice were anesthetized by ip ketamine (150 mg/kg) and acetylpromazine (15 mg/kg). Once surgical anesthesia was established, a tracheotomy was performed and a 19-gauge stainless steel cannula was inserted into the trachea. Animals were then paralyzed with pancuronium bromide (4 mg/kg, ip) and placed on a 37°C heating pad, where the cannula was connected to a computer-controlled ventilator. Ventilation was maintained at a rate of 120 breaths/min and a tidal volume of 9 mL/kg, with the heart rate monitored (PowerLab System, AD Instruments, Colorado Springs, CO) to ensure proper anesthetic depth. ACh (1–10 mg/kg) was injected into the inferior vena cava, and changes in airway pressure were recorded for 5 min, followed by calculation of the relative airway pressure time index (APTI)—the percentage increase of APTI within 5 min—to quantify airway responsiveness induced by ACh in various animal groups (Ewart et al. 1996; Grinnan et al. 2006; Wills-Karp et al. 1998).

### Bronchoalveolar lavage

We performed BAL by flushing the lungs with 1 mL cold Hanks balanced salt solution (HBSS; Invitrogen, Grand Island, NY) through the tracheal cannula, as previously described (Walters et al. 2002). The recovered lavage fluid (~ 0.7 mL) was centrifuged (500 × *g* for 20 min), and the cell pellet was resuspended in 200 μL ice-cold HBSS. Total cells were counted with a hemocytometer. Slides were prepared by cytocentrifugation (Cytospin 3; Shandon Instruments, Pittsburgh, PA) and stained with Diff-Quik (Dade Behring, Düdingen, Switzerland). We determined BAL cell differential counts using morphologic criteria under a light microscope with evaluation of 200 cells/slide. The supernatant from BAL fluid was centrifuged again (15,000 × *g* for 10 min), and the supernatant was stored at –80°C for further protein and cytokine analysis.

### Measurements of BAL proteins and cytokines

We measured protein concentrations in BAL fluid using the RC DC Protein Assay (Bio-Rad, Hercules, CA) according to the manufacturer’s recommendations. We converted optical density readings of samples to milligrams per milliliter using values obtained from a standard curve generated with serial dilutions of bovine serum albumin (0.1–1.5 mg/mL). Interleukin-4 (IL-4), IL-5, eotaxin, interferon-γ (IFN-γ), and tumor necrosis factor-α (TNF-α) were measured in unconcentrated BAL fluid using the BioPlex system as described by de Jager et al. (2003). Optical density readings of samples were converted to relative levels using values obtained from standard curves generated with serial dilutions of each recombinant cytokine.

### Lung histopathology

Murine lungs were excised and immersed in 10% formalin for at least 48 hr. The left lobe was removed and washed with 70% ethanol, dehydrated, and embedded in glycol methacrylate. Sections, cut 5–6 μm thick, were stained with hematoxylin and eosin (H&E); we examined two sections from each sample for evidence of inflammation, injury, and number of mucus-containing cells using Periodic Acid-Schiff (PAS) staining kit (Sigma Chemical Co., St. Louis, MO).

### RNA isolation and transcript analysis

We extracted total RNA from frozen lungs with a combined protocol using TRIzol reagent (Invitrogen, Carlsbad, CA) and the RNeasy kit (Qiagen, Valencia, CA) as previously described (Nonas et al. 2007; Simon et al. 2006). Frozen lungs were mixed with TRIzol reagent and homogenized with a Polyclone tissue homogenizer (Kinematica, Bohemia, NY). We used an RNeasy kit to further clean the total RNA extracted by TRIzol reagent. We routinely performed an on-column DNase I digestion. For each group, we prepared RNA from the lungs of three animals.

We used total RNA (5 μg) to synthesize double-stranded cDNA using the One-Cycle cDNA Synthesis Kit (Affymetrix, Santa Clara, CA). The cDNA served as a template to synthesize biotin-labeled antisense cRNA using an IVT Labeling Kit (Affymetrix). Labeled cRNA was fragmented and hybridized to the Affymetrix Mouse Genome 430 2.0 Array (containing ~ 34,000 genes), as described in the Affymetrix GeneChip protocol. Chips were scanned using a GeneChip Scanner 3000 (Affymetrix).

### Oligonucleotide array analysis

We evaluated chip quality, including RNA degradation, reverse transcription, cRNA synthesis and labeling, hybridization, chip washing, and scanning, uing GCOS, dChip (Li and Hung Wong 2001), and the Bioconductor Affy package (Bolstad et al. 2003). All RNA samples and chips used in this study passed established quality criteria (data not shown). We calculated the intensities of probe sets using the gcrma package of Bioconductor software (R Development Core Team 2005) with GC robust multichip average (GCRMA) normalization (Wu et al. 2004). To identify differentially expressed genes, we conducted two-group comparison using Significance Analysis of Microarrays (SAM) (Chu et al. 2003). We defined “dys-regulated genes” as the differentially expressed genes identified when a normal control group was used in a pairwise comparison. Only the probe sets that were present (determined by Affy P-call) in all three replicates of at least one group in the pairwise comparison were used for data analysis. The gene-filtering parameters and results are summarized in Supplemental Material, Table 1 (http://www.ehponline.org/members/2008/11229/suppl.pdf). For probe sets representing the same Entrez Gene [National Center for Biotechnology Information (NCBI) 2008a] or UniGene accession numbers (NCBI 2008c), we included only the probe set with the lowest false detection rate (FDR) or the highest fold changes in the gene list. The microarray data have been submitted to the NCBI’s Gene Expression Omnibus (GEO) Datasets (NCBI 2008b) (GSE9465).

### Identification of Gene Ontology (GO) categories enriched with dysregulated genes

The functional profiles were represented by the biological processes in the GO database (Gene Ontology Consortium 2006). We compared the number of dysregulated genes in each GO category with that of all genes in the Affymetrix Mouse Genome 430 2.0 Array to determine the significance of the GO category. We performed the analysis using Onto-Express (the Gene Ontology 2008), with the default selection of statistic method (hypergeometric distribution followed by false discovery rate correction). We uploaded the lists of dys-regulated genes into Onto-Express to identify significant GO categories (the FDR-adjusted *p*-value, *q* ≤0.05 with six or more genes).

### Ingenuity pathway analysis (IPA)

We then uploaded the dysregulated genes into Ingenuity Pathways Analysis (IPA) software (Ingenuity Systems Inc. 2008). This Web-delivered application makes use of the Ingenuity Pathways Knowledge Base (IPKB) containing a large amount of individually modeled relationships between gene objects (e.g., genes, mRNAs, and proteins) in order to dynamically generate significant regulatory and signaling networks or pathways. The submitted genes that it maps to the corresponding gene objects in the IPKB are called “focus genes.” The significance of a canonical pathway is controlled by *p*-value, which is calculated using the right-tailed (referring to the overrepresented pathway) Fisher exact test for 2 × 2 contingency tables. This is done by comparing the number of “focus” genes that participate in a given pathway, relative to the total number of occurrences of those genes in all pathways stored in the IPKB. The significance threshold of a canonical pathway is set to 1.3, which is derived by –log_10_ (*p*-value), with *p* ≤0.05.

### Assessment of gene expression synergy in PM- and OVA-challenged mice

We employed a “synergy,” which we defined as the presence of the effects by PM plus OVA that was greater than the effects induced by PM or OVA alone and greater than the sum of those individual effects (Gong et al. 2007). We used a synergistic index,


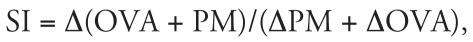


where Δ(OVA + PM) was the difference between levels of PBS/PBS and OVA/PM groups, ΔPM was the difference in levels between PBS/PM and PBS/PBS groups, and ΔOVA was the difference in levels between OVA/PBS and PBS/PBS groups. Synergy in physiologic parameters required the concurrence of three criteria: SI > 1, Δ(OVA + PM) > mean ΔPM (*p* < 0.05), and Δ(OVA + PM) > mean ΔOVA (*p* < 0.05).

### Nonmicroarray-related statistical analyses

Data are presented as group means ± SE. We performed statistical comparisons among treatment groups by randomized-design two-way analysis of variance followed by the Newman-Keuls post hoc test for more than two groups, or by an unpaired Student’s *t*-test for two groups. In all cases, we defined statistical significance as *p* < 0.05.

## Results

### PM-induced AHR in control and asthmatic mice

**Figure 1:**
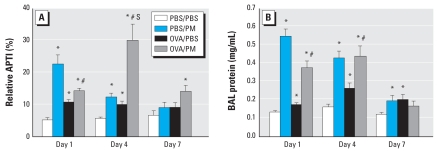
Effect of Baltimore PM on AHR (*A*) and BAL protein (*B*) in naive and OVA-sensitized mice. Values shown are mean ± SE (*n* = 5–6). (*A*) AHR, assessed by response to intravenously administered ACh (1 mg/kg), was increased on day 1 and remained elevated at least 7 days after PM exposure. PM stimulated significant AHR in OVA-challenged mice compared with PBS/PBS control A/J mice. Airway responsiveness is expressed as percentage of the time-integrated change in airway pressure over baseline pressure (APTI). *Significant increase over PBS/PBS control for each time point (*p* < 0.05). ^#^Significant change in response to PBS/OVA group for each time point (*p* < 0.05). S, synergistic effect between PM and OVA treatment (SI > 1).

To assess the contribution of ambient Baltimore PM to murine airway inflammation and asthmatic parameters, we used a well-established murine asthma model and assessed AHR as an indirect parameter of airway bronchoconstriction in response to the endogenous bronchoconstrictor ACh (Levitt and Mitzner 1988). We measured airway pressure changes stimulated by exogenously infused ACh to represent airway responses expressed as APTI, a widely used parameter to quantify AHR (Ewart et al. 1995, 1996; Grinnan et al. 2006). We determined that OVA challenge increased AHR in A/J mice on day 1 (1.9-fold increase) and day 4 (1.7-fold increase). AHR remained elevated on day 7 (1.8-fold increase), but was not statistically significant (Figure 1A). Similarly, PM exposure induced a prominent (4.0-fold) increase in AHR in naive A/J mice (PBS controls) on day 1, which waned over the ensuing week, returning to control levels by day 7. When we evaluated the effect of PM exposure on AHR in OVA-challenged mice with the established asthmatic phenotype, a marked synergy in AHR was maximal on day 4 after PM exposure (4.9-fold increase) (Figure 1A). By day 7, unlike either OVA or PM treatment alone, PM-mediated AHR in OVA-treated mice remained significantly elevated compared with PBS-challenged naive A/J mice (2.1-fold increase).

### PM-induced alveolar protein leakage

We assessed PM-mediated increases in BAL protein level as an indication of epithelial/endothelial barrier dysfunction and vascular leakage, as well as a key parameter of inflammatory lung injury. PM produced significant increases in the level of BAL protein on days 1 and 4 (4.4- and 2.7-fold increase, respectively) in naive A/J mice, with values declining to baseline levels at day 7 after PM exposure (Figure 1B). As expected, OVA challenge induced a mild increase in BAL protein content on days 1 and 4 (1.3- and 1.7-fold increase, respectively). Similar to PM effects in naive mice, PM induced increases in BAL protein in OVA-challenged A/J mice on days 1 and 4 (> 2-fold increase), with the increased BAL protein levels returning to basal concentrations on day 7 after PM challenge (Figure 1B).

### PM-induced inflammatory leukocyte infiltration into airways

**Figure 2:**
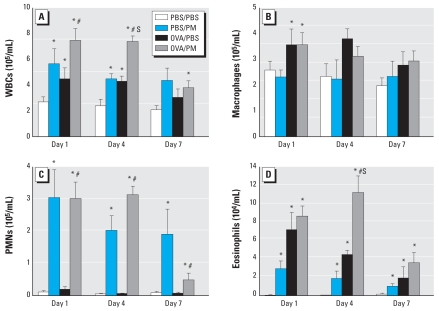
Effect of Baltimore PM on total BAL leukocyte counts. (*A*) Total white blood cells (WBCs). (*B*) Macrophages. (*C*) Neutrophils (polymorphonuclear cells; PMNs). (*D*) Eosinophils. Values shown are mean ± SE; *n* = 5–6. *Significant increase over PBS/PBS control for each time point (*p* < 0.05). ^#^Significant increase over PBS/OVA group for each time point (*p* < 0.05). S, synergistic effect between PM and OVA treatment (SI > 1).

PM has been reported to cause inflammatory leukocyte infiltration into airways and alveoli in various animal models (Dick et al. 2003; Kodavanti et al. 1999; Prahalad et al. 1999; Salvi et al. 2000; Walters et al. 2001). We assessed the effect of Baltimore PM on BAL leukocyte counts in both naive and OVA-challenged asthmatic mice (Figure 2A). PM induced a peak increase in total leukocyte count in BAL fluid on day 1 (2.5-fold increase) and remained significantly elevated on day 7. OVA reliably induced an increase in leukocytes on day 1 (1.6-fold increase), and this induction declined back to control levels by day 7. The combination of PM challenge in OVA mice, however, induced strong synergy in leukocyte infiltration into airways, which peaked on day 1 (Figure 2A) but was still highly increased on day 4 and remained elevated even on day 7.

We next analyzed the differential leukocyte types in the extracted BAL. PM induced significant eosinophil (days 1 and 4) and neutrophil (days 1, 4, and 7) infiltration into BAL without any changes in BAL macrophage counts. In contrast, OVA-sensitized mice exhibited increased numbers of macrophages and eosinophils at all three time points (Figure 2), consistent with reported findings (Kung et al. 1994; Nakajima et al. 1992). Combined OVA and Baltimore PM exposure was similar to OVA exposure alone except for a marked eosinophil infiltration into the airways on day 4, which was greater than either exposure alone (Figure 2).

### PM-stimulated T_H_1/T_H_2 cytokine secretion

**Figure 3:**
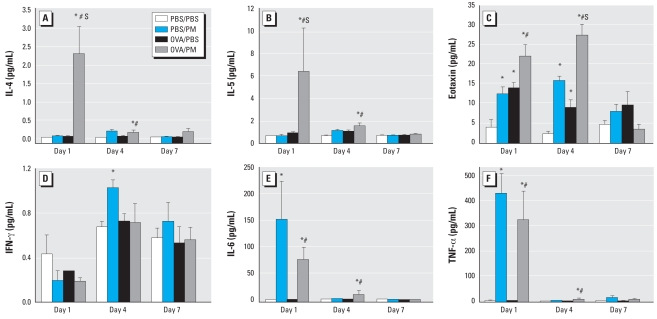
Effect of Baltimore PM on T_H_2 (*A–C*) and T_H_1 (*D*–*F*) cytokine levels in BAL fluid. (*A–C*) Levels of BAL T_H_2 cytokines IL-4 (*A*), IL-5 (*B*), and eotaxin (*C*) and (*D*–*F*) levels of BAL T_H_1 cytokines IFN-γ (*D*), IL-6 (*E*), and TNF-α (*F*). Values are mean ± SE; *n* = 4–5. *Significant increase over PBS/PBS control for each time point (*p* < 0.05). ^#^Significant increase over PBS/OVA group for each time point (*p* < 0.05). S, synergistic effect between PM and OVA treatment (SI > 1).

We next examined the levels of T_H_1 cytokines (IL-6, IFN-γ, TNF-α) and T_H_2 cytokines (IL-4, IL-5, eotaxin) in BAL fluid (Figure 3) after PM and OVA challenge. Levels of IL-4 and IL-5 were not significantly altered by direct PM exposure or OVA challenge. In contrast, marked increases in IL-4 and IL-5 were produced by the combined challenge of OVA and PM, results that were maximal at day 1 after PM treatment (35- and 9-fold, respectively) and remained significantly elevated through day 4 (3.1- and 2.1-fold, respectively). Levels of the eosinophil chemo-attractant eotaxin are known to be increased during eosinophil infiltration and T_H_2 type inflammation (Mattes and Foster 2003; Pease and Williams 2001). Predictably, and consistent with marked eosinophil recruitment, OVA challenge increased eotaxin levels in BAL (days 1 and 4). Despite only a modest effect on BAL eosinophil content, PM increased eotaxin levels in the BAL of naive mice and was markedly synergistic in OVA-challenged asthmatic mice (days 1 and 4). The effects of PM decreased to baseline on day 7 after PM challenge.

Several types of PM are known to stimulate secretion of the proinflammatory cytokines IL-6 and TNF-α (Dick et al. 2003; Walters et al. 2001). We assayed T_H_1 cytokines (IFN-α, IL-6, TNF-α) in BAL fluid and discovered a strong PM-mediated T_H_1-type inflammatory profile with active induction of IL-6 and TNF-α(days 1 and 4) with a minimal effect on IFN except on day 4 (Figure 3D–F). OVA challenge did not affect T_H_1 cytokine secretion at any time point, and we identified no synergism between PM and OVA that elevated these T_H_1 inflammatory cytokines.

### PM-induced histologic alterations

**Figure 4:**
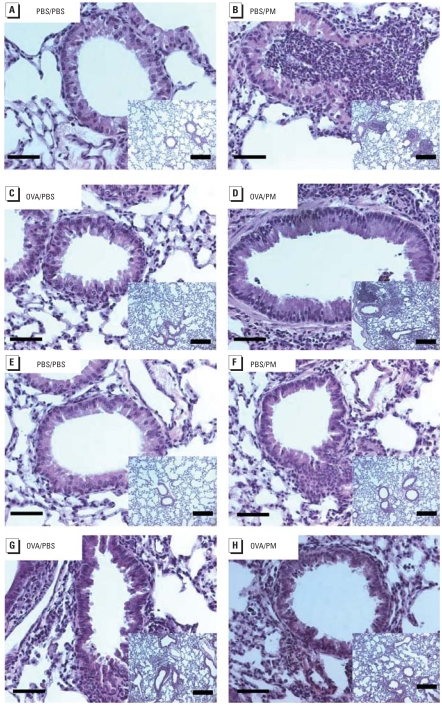
Effect of Baltimore PM on histologic alterations in the lung on days 1 (*A–D*) and 7 (*E–H*) after treatment with Baltimore-PM. (*A,E*) PBS/PBS; (*B,F*) PBS/PM; (*C,G*) OVA/PBS; (*D,H*) OVA/PM. PM induced inflammatory leukocyte infiltration in both control mice (*A*) and OVA-challenged mice (*D*). PM residues are retained in alveoli. At 7 days, leukocyte infiltration declined to basal level (*F, H*). Tissues were stained with H&E; each panel is representative of paraffin sections from three A/J mice with the same treatment. Bars = 50 μm; bars in insets = 300 μm.

**Figure 5:**
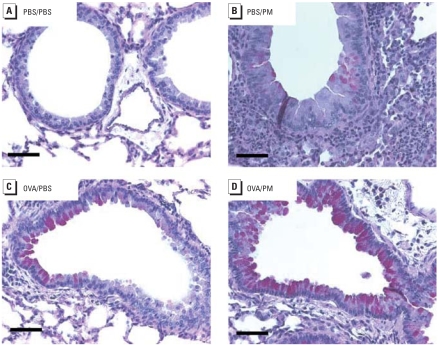
Effects of PM-induced mucus secretion in murine airways. Paraffin sections of the lung were stained with periodic acid-Schiff (PAS) to detect presence of goblet cells and activity at 4 days after Baltimore PM treatment. (*A*) PBS/PBS; (*B*) PBS/PM; (*C*) OVA/PBS; (*D*) OVA/PM. Each panel is representative of paraffin sections from three A/J mice in the same treatment group. PM or OVA induced PAS-positive purple staining. PM and OVA induced PAS-positive purple staining synergistically. Bars = 50 μm.

We next confirmed the inflammatory changes observed in lung tissue sections subjected to H&E staining after PM challenge in naive and OVA-challenged mice (Figure 4). We observed inflammatory leukocyte infiltration into vessels, airways, and alveoli after OVA or PM treatment on day 1 after treatment (Figure 4B–D). PM also induced leukocyte infiltration in OVA-challenged asthmatic mice. By day 7 (Figure 4E–H), the amount of infiltrated cells declined in PM-treated mice (Figure 4F,H), consistent with the BAL cellularity analysis (Figure 2A) and cytokine profiles (Figure 3). On day 1, we observed conjugated PM pellets deposited in alveoli, reflecting PM exposure (Figure 4B), but they were gradually eliminated from the lung and became undetectable after 7 days (data not shown). PM also induced mucus secretion into the airways (Figure 5). PM, as well as OVA, activated mucus-producing goblet cells in murine airways day 4 post-challenge. PM exhibited a stronger impact on mucus activation in the OVA-remodeled airways. Although leukocyte infiltration declined 7 days after PM exposure, elevated mucus secretion persisted until day 7 (data not shown).

### Differential lung gene expression by PM or OVA challenge

We next used the Affymetrix platform (Mouse Genome 430 2.0 Array) to profile changes in gene expression after the intratracheal installation of Baltimore PM in naive and OVA-challenged mice. PM had a strong impact on the global expression of lung genes, with > 1,200 genes differentially regulated by PM (*p* < 0.05) on day 4 after exposure. With extremely stringent conditions (FDR < 0.3% and fold change > 3-fold), 436 genes survived filtering and were identified as significantly dysregulated by PM exposure (375 genes up-regulated and 61 genes down-regulated). In contrast, OVA challenge had less impact on lung gene expression than did PM at day 4 after exposure. Only 37 genes (21 genes up-regulated and 16 genes down-regulated) were differentially regulated by OVA sensitization even when less stringent criteria (FDR < 5% and fold change > 2-fold) were applied. The combination of PM and OVA treatment exhibited synergistic effects on lung gene expression, with a total of 591 genes identified as differentially regulated [492 genes up-regulated and 99 genes down-regulated (FDR < 0.3% and fold change > 3-fold)].

The PM-regulated genes were related to 22 biological processes, including innate immune response, chemotaxis, cell-surface receptor–linked signal transduction, inflammatory response, defense response, cell cycle, nervous system development, and DNA-dependent regulation of transcription (Table 1). Of the 436 PM-regulated genes, we defined 25 genes as the most differentially regulated (fold change > 25-fold; Supplemental Material Table 1 available online at http://www.ehponline.org/members/2008/11229/suppl.pdf). Interestingly, 17 of these 25 genes were closely linked with asthma/airway inflammation, immune responses, chemokine/ cytokine, inflammation, and epithelial cell proliferation (Supplemental Material, Table 2 available online at http://www.ehponline.org/members/2008/11229/suppl.pdf). These data indicate that PM produces profound lung inflammation in a manner that contributes to asthma phenotypes, such as inflammatory leukocytes maturation, cytokine secretion, and airway remodeling.

**Figure 6:**
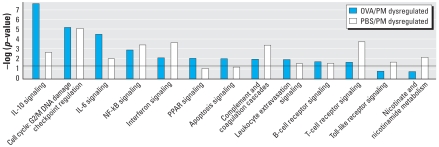
Biological processes detected by IPA of OVA/PM and PBS/PM dysregulated genes. Abbreviations: NF-κB, nuclear factor-κB; PPAR, peroxisome proliferator-activated receptor. Several of the top significant canonical pathways were enriched with dysregulated genes induced by PM or OVA/PM treatment. The horizontal line indicates the threshold. See Supplemental Material, Table 2 (online at http://www.ehponline.org/members/2008/11229/suppl.pdf) for the description of gene selections with SAM software.

IPA analyzes the pathways generated by differentially expressed genes in a pairwise fashion compared with either lung gene dysregulation with PM or the combined PM and OVA challenge (Figure 6). Similar to GO analysis, cell cycle, inflammatory response (interleukin signaling, IFN signaling), and cell-surface receptor (B-cell receptor, T-cell receptor, and Toll-like receptor) pathways were among the most distinctly regulated pathways. Most of these signaling pathways are closely related to asthma development. For example, the genes in the complement system were significantly regulated by PM in both the control (PM group) and the asthma animals (PM and OVA group). These genes are implicated in the development of asthmatic phenotypes (Wills-Karp 2007).

### Regulation of asthmatic genes by PM

**Figure 7:**
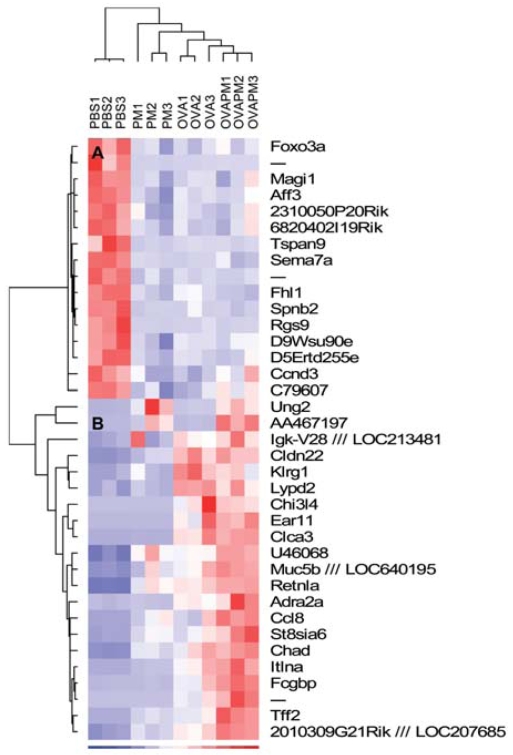
Hierarchical clustering of OVA-induced dysregulated genes by dChip. The 37 dysregulated genes induced by OVA treatment were selected by SAM software (see Supplemental Material, Table 1, online at http://www.ehponline.org/members/2008/11229/suppl.pdf). Sample clustering is displayed at the top; the two gene clusters A and B are displayed on the left. Blue, white, and red represent the expression level below, at, and above mean level, respectively. PBS1, PBS control sample 1; PM1, PM-treated sample 1; OVA1, OVA-treated sample 1; OVAPM1, OVA- and PM-treated sample 1.

The expression profiles of the most differentially regulated asthma genes (by OVA) across all samples were next displayed in dChip (Figure 7). Most of the OVA-regulated genes were consistent with previously reported data (Izuhara and Saito 2006; Kuperman et al. 2005). Hierarchical clustering on individual samples correctly classified replicates into the corresponding experimental group, indicating that the OVA-regulated genes displayed a differential expression profile in each experimental condition (PM, OVA, PM/OVA). We also divided samples into two further clusters: one with only PBS control samples and the second containing all OVA, PM, and OVA/PM treated samples. We grouped expression levels of genes into two primary clusters: a down-regulated gene cluster and an up-regulated gene cluster (Figure 7). In the cluster of down-regulated genes, PM and OVA challenge and the combined exposure exhibited the same trend of gene down-regulation, whereas in the up-regulated cluster of genes, most genes exhibited an additive or synergistic pattern with both PM and OVA exposures leading to an exacerbation of asthmatic gene expression by PM in OVA-sensitized animals. Of all OVA regulated genes, 14 of 37 genes were identified in the SAM list of PM dysregulated genes, including *It1na* (interlectin), a typical marker of airway inflammation; *Tff2* (trefoil factor 2), a marker gene for airway mucus generation and asthmatic phenotypes; and *Clca3* (chloride channel 3), a well-recognized marker gene of mucus formation, airway inflammation, and AHR (Nakanishi et al. 2001; Nikolaidis et al. 2006) (Table 2). *It1na* (SI > 1.52), *Tff2* (SI > 1.60), and *Clca3* (SI > 1.16) were all up-regulated by PM and OVA synergistically.

## Discussion

Epidemiologic studies have firmly linked increased ambient PM exposure to increased cardiopulmonary morbidity and mortality (Samet and Krewski 2007), including exacerbation of preexisting conditions such as asthma and COPD. Our present results, using ambient PM from Baltimore, confirm and extend previous studies that PM exposure induces asthmalike parameters of AHR and airway inflammation defined by increases in BAL protein, eosinophils, and neutrophils. Current concepts of asthma pathobiology strongly suggest that these factors contribute to asthma symptoms (Walters et al. 2001, 2002) and stimulate secretion of asthma-producing T_H_2 cytokines (IL-5 and eotaxin) into BAL (Walters et al. 2001). To extend prior findings beyond the biological effects of PM in naive mice, we used an established OVA-challenged murine asthma model (Archer et al. 2004; Fernvik et al. 2002; Handzel 2000; Lambert et al. 1999; Walters et al. 2001) and assessed potential synergy between PM exposure and the established asthmatic phenotype at several time points after PM exposure. In addition, we applied extensive biochemical and genomic strategies to mechanistically assess PM-mediated asthma pathobiology. We now report the direct induction of asthma-related parameters by PM and PM-mediated exacerbation of preexisting murine asthma parameters, in association with a definable molecular signature composed of differentially regulated genes relevant to asthma pathogenesis.

Ambient particles are classified for health purposes by aerodynamic diameter, as follows: coarse [< 10 μm (PM_10_)], fine (< 2.5 μm (PM_2.5_)], and ultrafine/nanoparticles [< 0.1 μm (PM_0.1_)]. We used a single Baltimore PM sample with a median diameter of 1.78 μm, which allows adequate upper and lower respiratory tract deposition (Figure 4B,D) when delivered via intratracheal instillation (Walters et al. 2001, 2002). This intratracheal administration mode is not representative of physiologic conditions and represents a high dose rate. This single administered dose is equivalent to an exposure over a period of months. We recognize potential limitations with this approach.

A key finding in the present study is the demonstration that PM not only induces AHR in naive mice but also is synergistic in exacerbating AHR in mice with preexisting enhanced AHR (OVA-sensitized mice 4 days after PM exposure). Because diesel exhaust particles, a major source of Baltimore PM, are recognized as adjuvants during allergen exposure (Nel et al. 1998; Ohta et al. 1999; Porter et al. 2007; Walters et al. 2001), the possibility that ambient PM is generally involved in the increased prevalence of human atopic asthma is highly plausible. We did not directly address the hypothesis that PM contributes to asthma susceptibility; however, our data collected from mice with established increased AHR indicate that PM significantly increases the severity and duration of several features observed during acute asthma exacerbations (Walters et al. 2001). The findings include increased BAL eosinophils and BAL eotaxin and IL-5 generation, suggesting that PM directly affects AHR independently of an effect on allergic sensitization. In addition to increased AHR, we observed that PM exposure induces increases in BAL protein, reflecting increases in vascular and epithelial permeability, a cardinal feature of the inflammatory response likely related to elevated levels of PM-containing reactive oxygen species (Gualtieri et al. 2007; Li et al. 2003) or to PM-mediated recruitment of leukocytes to challenged airways.

Limited information exists concerning the toxicogenomic effects of PM on lung tissue, a viable strategy for the identification of potential molecular biomarkers that may reflect the pulmonary toxicity of PM exposure (Andre et al. 2006; Koike et al. 2004; Kooter et al. 2005; Leikauf et al. 2001; Roberts et al. 2004; Wise et al. 2006). Because asthmalike parameters peaked 3–7 days after PM treatment (Walters et al. 2001), we chose day 4 after PM exposure as the time point to assess the impact of PM on lung tissue genome regulation, a time point of maximal synergy with OVA sensitization. The PM impact on global gene expression was extremely strong, despite extremely stringent conditions, with most of the 436 filtered genes representing biological processes closely associated with asthmatic parameters, such as immune responses, innate immune responses, inflammatory responses, and leukocyte chemotaxis. Although ambient particle-induced oxidative stress may contribute to inflammatory and toxic effects (Li and Nel 2006; MacNee and Donaldson 2003; Xia et al. 2007), we did not identify this gene ontology within the 436 genes in the SAM list.

Our genomic results (confirmed by enzyme-linked immunosorbent assay and reverse-transcriptase polymerase chain reaction approaches) show the previously described propensity for PM to induce a strong pro-inflammatory molecular signature, as well as a strong genomic signature involving activation of biological pathways linked to the development of asthmatic phenotypes, such as the induction of the modulation and secretion of key T_H_2 cytokines (e.g., IL-4, IL-5, eotaxin). Eosinophilic inflammation is a hallmark of asthma, and exposure to Baltimore PM was a potent stimulus for an influx of eosinophils (as well as neutrophils) into the murine lung (Figures 1B, 2, 4) and synergistically induced eosinophil infiltration in asthmatic mice, likely major contributors to the sustained AHR response in PM-stimulated OVA-sensitized mice. IL-4, a cytokine with marked PM-mediated increases in gene and protein expression, regulates allergic inflammation by eosinophil adhesion and recruitment to lung airways, promotes T_H_2 cell differentiation, and direct stimulates airway remodeling (Kips 2001). Both IL-5, a primary cytokine involved in eosinophil differentiation, maturation, and activation (Hogan et al. 1998), and eotaxin, an eosinophil chemotaxin (Conroy and Williams 2001), exhibited markedly up-regulated expression by PM in asthmatic mice. These results provide a viable mechanism for PM contribution to asthma development and propensity for asthmatic exacerbations.

PM-mediated gene expression overlaps significantly with many OVA-driven genes identified in our model of murine asthma with expression in exactly the same direction (often with significant synergy); that is, PM down-regulates all OVA–down-regulated genes and up-regulates all OVA–up-regulated genes (Figure 7). OVA induces 37 differentially expressed genes, with 14 of these genes also listed within the PM-regulated SAM list (Table 2) including the potent asthma bio-markers *Clca3* and *Tff2* (Nikolaidis et al. 2006). *Clca3* encodes a calcium-activated chloride channel found to be critical in mucus overproduction and AHR in asthma (Nakanishi et al. 2001). The T_H_2 cytokines IL-4 and IL-13 induce *Clca3* expression in epithelium in association with goblet cell metaplasia, mucus overproduction, and augmented allergic airway responses (Berlin et al. 2004). Trefoil factor-2 (*Tff2*) is an allergen-induced gene regulated by T_H_2 cytokines in the lung that promotes human bronchial epithelial cell migration and formation of mucus-producing airway cells (Nikolaidis et al. 2006). Consistent with the dramatic up-regulation of genes that encode factors that affect goblet cell activity and mucus production, such as *Tff2*, *Clca3*, and *Muc5b*, another marker for mucus stimulation, PM exhibited prominent effects on mucus stimulation as indicated by periodic acid-Schiff (PAS) staining (Figure 5) in both control and OVA-challenged mice.

Analysis of the gene ontologies stimulated by PM in naive mice demonstrated similarities to prior PM-related genomic studies with the induction of airway inflammation activity. For example, acute exposure (4–24 hr) of ultrafine PM induced expression of genes involved in oxidation stress, inflammation, transcription regulation, and cardiovascular-related function without a link to asthma development and exacerbation (Andre et al. 2006; Kooter et al. 2005). IL-6 and TNF-α are proinflammatory cytokines that regulate innate immunity physiologic host defense processes during infection, airway damage in COPD, and acute lung injury (Chung 2001). Unlike results in OVA-challenged mice alone, PM stimulates the generation and secretion of IL-6 and TNF-α into airways (Figure 4) with marked neutrophil infiltration. Another key PM signature in naive mice is involvement of complement activation pathways. Complement factors C3 and C5 are potent chemoattractants for inflammatory cells (neutrophils and eosinophils) and anaphylatoxins, which trigger smooth muscle contraction and regulate vasodilation (Bolger et al. 2007). C3 and C5 are also known to increase vascular permeability, a clear phenotypic parameter in PM-challenged OVA mice where PM exposure induces significant changes in vascular permeability as assessed by protein content in BAL fluids (Figure 2A). Complement factor B is a key regulator in the development of AHR and inflammation (Taube et al. 2006). In addition, we identified complement factor 3 to mediate PM-induced AHR, indicating that AHR induced by PM depends on C3 activation in the airways (Walters et al. 2002). Consistent with a role in mediating PM-regulated airway function, we found five genes from the complement system family that are differentially affected by PM: complement component 1, q subcomponent, alpha poly-peptide (3.0-fold); complement component 1, q subcomponent, beta polypeptide (3.2-fold); complement component 1, q subcomponent, C chain complement (3.5-fold); component 3a receptor 1 (35-fold); and complement factor B (17-fold).

Our genomic approaches also allowed us to identify multiple, potentially novel, biomarkers for development of asthmatic lung responses induced by PM. *Rgs9* is a family member of regulators of G-protein signaling that act as GTPase-activating–proteins specific to the Gα subunit. It plays a critical role in the termination process of G-protein–mediated cell responses in eukaryotes (Hepler 1999). *Rgs9* in retinal photo-receptor cells induces an elevation in local cyclic guanosine monophosphate (cGMP) concentration and closes cGMP-gated cation channels (Arshavsky et al. 2002). PM reduced *Rgs9* transcription in lung tissues (6.2-fold), whereas OVA had a similar effect (5.2-fold). Our finding that *Rgs9* is highly transcribed in lung tissues is novel, although a localized function has not yet been elucidated. Intelectin is an intestinal antimicrobial factor (Datta et al. 2005) induced by IL-13 in human airway epithelial cells (Kuperman et al. 2005). The molecular patterns recognized by intelectin include furanosides such as galactofuranose and galactofuranosyl residues that are present in bacterial and fungal cell walls and in protozoan parasites, but not in mammalian cells. Intelectin was markedly induced by PM (35-fold increase) and represents another potential signature of PM-induced lung toxicity. Although intelectin in the airway may serve as a novel defensive gene altering the response of asthmatics to infection, the contribution of intelectin to asthma pathogenesis requires further exploration.

In summary, exposure of a murine preclinical model of asthma to urban PM results in elevation of AHR, BAL protein leakage, inflammatory leukocyte infiltration, and T_H_2/T_H_1 cytokine secretion. Baltimore PM also produced a strong molecular signature composed of inflammatory/asthmatic gene expression in naive mice, with marked synergy with OVA-sensitized mice. These studies are consistent with emerging epidemiologic evidence and indicate that PM exposure evokes proinflammatory and allergic molecular signatures that may directly contribute to the asthma susceptibility in naive subjects and increased severity in affected asthmatics.

## Figures and Tables

**Table 1:** Biological process enriched with dysregulated genes induced by OVA/PM.^a^

GO ID	Function name	Gene	*q*-Value
GO:0000074	Regulation of progression through cell cycle	13	3.22 × 10^−3^
GO:0006260	DNA replication	12	3.84 × 10^−5^
GO:0006355	Regulation of transcription, DNA-dependent	28	4.05 × 10^−2^
GO:0006468	Protein amino acid phosphorylation	22	4.31 × 10^−2^
GO:0006508	Proteolysis	26	6.59 × 10^−4^
GO:0006811	Ion transport	21	1.94 × 10^−2^
GO:0006817	Phosphate transport	6	8.36 × 10^−3^
GO:0006911	Phagocytosis, engulfment	6	1.63 × 10^−7^
GO:0006935	Chemotaxis	18	1.13 × 10^−10^
GO:0006952	Defense response	12	3.60 × 10^−4^
GO:0006954	Inflammatory response	34	1.56 × 10^−10^
GO:0006955	Immune response	57	2.11 × 10^−10^
GO:0006974	Response to DNA damage stimulus	10	2.37 × 10^−2^
GO:0007049	Cell cycle	49	2.37 × 10^−10^
GO:0007067	Mitosis	31	2.14 × 10^−10^
GO:0007155	Cell adhesion	23	1.37 × 10^−3^
GO:0007165	Signal transduction	41	2.59 × 10^−4^
GO:0007166	Cell surface receptor linked signal transduction	9	8.33 × 10^−4^
GO:0007242	Intracellular signaling cascade	17	2.56 × 10^−2^
GO:0008283	Cell proliferation	7	3.13 × 10^−2^
GO:0045087	Innate immune response	10	2.04 × 10^−6^
GO:0051301	Cell division	40	2.16 × 10^−10^

a GO categories identified by Onto-Express software; only biological processes with more than five genes and *q*-values < 0.05 are shown (see “Materials and Methods” for details).

**Table 2:** Intersection between PM- and OVA-induced dysregulated genes.

Probe set ID	Symbol	OVA	PM	GO category
1439423_x_at	*U46068*	2.8	3.2	Lipid binding
1419684_at	*Ccl8*	26.2	26.7	Chemotaxis
1416306_at	*Clca3*	3989.6	64.0	Chloride transport
1434046_at	*AA467197*	4.6	68.6	Other
1459003_at	*Fhl1*	0.3	0.2	Cell differentiation
1447918_x_at	*LOC207685*	7.4	3.2	Humoral immune response
1418165_at	*Itlna*	197.5	35.3	Signal transduction
1439635_at	*Rgs9*	0.2	0.2	G-protein coupled receptor protein signaling pathway
1449015_at	*Retnla*	16.2	13.4	Hormone activity
1422040_at	*Sema7a*	0.3	0.3	Multicellular organismal development
1456440_s_at	*St8sia6*	3.8	3.4	Protein amino acid glycosylation
1443361_at	*Tspan9*	0.3	0.2	Other
1422448_at	*Tff2*	20.3	12.3	Other
1455114_at	*Ung2*	2.2	11.8	Regulation of progression through cell cycle DNA repair

Fold changes of dysregulated genes selected by SAM software (Supplemental Material Table 2, online at http://www.ehponline.org/members/2008/11229/suppl.pdf). For all genes, *q* < 0.1%. Corresponding GenBank accession numbers are available from Affymetrix (https://www.affymetrix.com/analysis/netaffx/quickquery.affx?netaffx=netaffx4_annot) with probe ID.
